# A Feasibility Study into the Production of a Mussel Matrix Reference Material for the Cyanobacterial Toxins Microcystins and Nodularins

**DOI:** 10.3390/toxins15010027

**Published:** 2022-12-30

**Authors:** Andrew D. Turner, Daniel G. Beach, Amanda Foss, Ingunn A. Samdal, Kjersti L. E. Løvberg, Julia Waack, Christine Edwards, Linda A. Lawton, Karl J. Dean, Benjamin H. Maskrey, Adam M. Lewis

**Affiliations:** 1 Centre for Environment Fisheries and Aquaculture Science, Barrack Road, Weymouth DT4 8UB, UK; 2 Biotoxin Metrology, National Research Council Canada, Halifax, NS B3H 3Z1, Canada; 3 Greenwater Laboratories, 205 Zeagler Drive, Suite 302, Palatka, FL 32177, USA; 4 Norwegian Veterinary Institute, 1431 Ås, Norway; 5 CyanoSol, School of Pharmacy and Life Sciences, Robert Gordon University, Aberdeen AB10 7GJ, UK

**Keywords:** microcystins, nodularins, mussels, reference materials, LC-MS/MS, LC-HRMS, shellfish, quality control

## Abstract

Microcystins and nodularins, produced naturally by certain species of cyanobacteria, have been found to accumulate in aquatic foodstuffs such as fish and shellfish, resulting in a risk to the health of the seafood consumer. Monitoring of toxins in such organisms for risk management purposes requires the availability of certified matrix reference materials to aid method development, validation and routine quality assurance. This study consequently targeted the preparation of a mussel tissue reference material incurred with a range of microcystin analogues and nodularins. Nine targeted analogues were incorporated into the material as confirmed through liquid chromatography with tandem mass spectrometry (LC-MS/MS), with an additional 15 analogues detected using LC coupled to non-targeted high resolution mass spectrometry (LC-HRMS). Toxins in the reference material and additional source tissues were quantified using LC-MS/MS, two different enzyme-linked immunosorbent assay (ELISA) methods and with an oxidative-cleavage method quantifying 3-methoxy-2-methyl-4-phenylbutyric acid (MMPB). Correlations between the concentrations quantified using the different methods were variable, likely relating to differences in assay cross-reactivities and differences in the abilities of each method to detect bound toxins. A consensus concentration of total soluble toxins determined from the four independent test methods was 2425 ± 575 µg/kg wet weight. A mean 43 ± 9% of bound toxins were present in addition to the freely extractable soluble form (57 ± 9%). The reference material produced was homogenous and stable when stored in the freezer for six months without any post-production stabilization applied. Consequently, a cyanotoxin shellfish reference material has been produced which demonstrates the feasibility of developing certified seafood matrix reference materials for a large range of cyanotoxins and could provide a valuable future resource for cyanotoxin risk monitoring, management and mitigation.

## 1. Introduction

Cyanobacterial blooms occur globally, affecting humans and animals through contamination of water and foodstuffs with harmful toxins [1]. Toxin classes most frequently encountered are the cyclic microcystins (MCs) [2,3] the cyclic pentapeptides nodularins (NODs), cylindrospermopsin and the neurotoxins including anatoxins and saxitoxins [2,4,5,6]. MCs and cylindrospermopsin are usually primarily described as hepatotoxins, but there is also evidence linking these to neurotoxicity, cardiotoxicity, immunotoxicity and developmental toxicity in a wide range of species [7,8]. MCs are produced primarily by freshwater cyanobacteria genera such as *Microcystis*, *Planktothrix*, *Anabaena/Dolichospermum* and *Oscillatoria* [9] with at least 310 analogues reported in blooms, cultures and/or from biotransformation reactions in the cell, environment or during sample processing [10,11,12], with increasing reports of MCs found in the marine environment [13,14,15]. Nodularin-R (NOD-R), along with a low number of structural analogues, is produced by *Nodularia spumigena* which thrives in brackish water [16], thereby resulting in impacts in both freshwater and saline environments [17,18,19]. Human health impacts from cyanotoxins have occurred through drinking from contaminated water supplies, recreational exposure, aerosol exposure under certain meteorological conditions and through eating contaminated aquatic foodstuffs, health food supplements, or even terrestrial foods irrigated with contaminated water [17,18,19,20,21,22,23,24,25,26]. In recent years, the risk from cyanotoxin poisoning following human consumption of seafood contaminated with cyanotoxins has been highlighted, with shellfish being one of the primary routes of toxins through to higher trophic levels [6,13,14,27,28,29] and in some instances with toxin concentrations reaching dangerously high levels (reviewed by [6]). 

**Figure 1:**
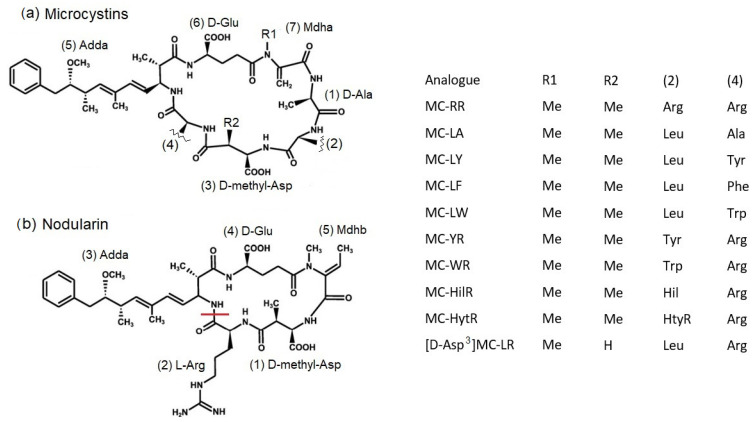
Generalized structure of (**a**) MCs and (**b**) NOD highlighting substituents present in analogues targeted in this study. Leu—Leucine, Arg—Arginine, Ala—Alanine, Tyr—Tyrosine, Hil—Homoisoleucine, Trp—Tryptophan, Phe—Phentylalanine, Me—Methyl, HtyR—Homotyrosine. Cyclic NOD-R structure shown, highlighting bond broken for hydrolysis to [seco-2/3] NOD (also known as linear nodularin).

Figure 1 illustrates the general structure of MCs and NODs, with both families containing the Adda moiety (3-amino-9-methoxy-2,6,8-trimethyl-10-phenyldeca-4(*E*),6(*E*)-dienoic acid [30]. The toxins act through inhibition of serine/threonine protein phosphatases resulting in cell damage and death, with acute symptoms including gastroenteritis, neurotoxicity, liver damage and potentially fatality [31,32]. Structural variations in the MCs include most notably variable L-amino acid residues at positions 2 and 4, as indicated by a two-letter suffix (e.g., MC-LR equating to L (leucine) and R (arginine)), although all other residues have been found to exhibit some variability as highlighted by a prefix to the name (e.g., [D-Asp^3^]MC-LR which contains D-aspartic acid at position 3) [11]. Although health impacts from cyanotoxins are found globally, few regulations exist in global legislation. The World Health Organization (WHO) has provided guideline values of 1 µg/L and 12 µg/L thresholds for lifetime and short-term exposure, respectively, to MC-LR in drinking waters [32,33].

Numerous approaches are published for the detection and quantitation of MCs and NODs [34], but with the majority to date focused primarily on water or cyanobacterial sample matrices [35,36]. The most common approaches include sensitive biochemical assays such as enzyme-linked immunosorbent assays (ELISA) [37] and protein phosphate inhibition assays (PPIA) [38], as well as instrumental chemical detection methods, primarily high-performance liquid chromatography with photodiode array detection (HPLC-PDA) [39], or LC coupled to mass spectrometry (LC-MS) [40,41,42]. Broadly speaking the biochemical assays are able to detect a wide range of structural analogues providing a quantitative assessment of total MC/NOD presence. Whilst PPIA methods show similar response for both Adda and non-Adda substituted MCs, the commercial Eurofins Abraxis ELISA is incapable of detecting the non Adda-substituted analogues, with cross-reactivities of <0.25% in comparison with MC-LR [37,43]. The low cross-reactivities determined for [ADMAdda^5^] and [DMAdda^5^]-MCs consequently evidences potential under-estimation of toxicity when conducting ELISA quantitation [37,43]. A multihapten ELISA based on polyclonal antibodies was, however, raised against a mixture of five MC analogues, resulting in wider cross-reactivities [44]. Instrumental detection methods typically target known compounds for which reference standards are available, although high-resolution mass spectrometry has also been applied for non-targeted analysis of other MC analogues unavailable as commercial standards [25,45,46], sometimes in combination with a thiol-derivatization approaches prior to analysis [47,48]. 

Due to the presence of a reactive α,β-unsaturated amide functional group in the Mdha^7^ amino acid common to most MCs, they form conjugates with thiol-containing biomolecules such as glutathione (GSH) and cysteine. Whilst LC-MS/MS can be used for direct measurement of these soluble small molecule conjugates [49,50,51,52], or potentially the parent toxins following deconjugation [18], most published instrumental methods focus solely on the free (soluble) toxins. However, there is the potential for conjugation with larger biomolecules in seafood tissue that are not readily extractable during sample preparation [52]. For example, non-extractable MCs are known to bind covalently to protein phosphatases and other thiol-containing proteins in tissues [53,54,55,56,57,58,59], so may potentially transfer to higher trophic levels. As such, assuming that covalently bound MCs are bioavailable and inhibit protein phosphatases after digestion [20,60,61], detection methods are required which can quantify both soluble and bound (total) toxins, noting the huge variability in proportions of soluble to bound toxins reported to date [58]. Consequently, whilst ELISA and LC-MS/MS results have been shown to compare well for algal and powdered algal product matrices [62,63], for biological tissue samples the ELISA may return higher quantitative results than targeted LC-MS/MS methods. This can be attributed to the MC antibodies used in the assays cross-reacting with the conjugates as well as the potential for MS-related ion suppression, if either matrix-matched calibrants, standard additional quantitation or isotope-dilution recovery correction are not applied [64,65]. 

A common approach to the analysis of total MCs and NODs combined (MCs + NODs) involves the use of a specific chemical reaction called the Lemieux oxidation. This reaction involves the catalyzed oxidative cleavage of an olefin to form two aldehydes or ketones. For MCs + NODs this reaction yields 3-methoxy-2-methyl-4-phenylbutyric acid (MMPB) from the Adda moiety within MCs and NOD-R, regardless of their initial amino acid configuration, which are then detected using mass spectrometric methods such as LC-MS/MS. This approach consequently provides a measure of total Adda-containing MCs + NODs [66,67], irrespective of form (soluble, bound or partially degraded [65]). This MMPB formation method has been used for this purpose in a variety of sample matrices including canine tissues [65] and shellfish [58,68] as well as enzymatic hydrolysis to form soluble microcystin-conjugated peptides [69], although these irreversible reactions result in the loss of information regarding original MC profiles. Furthermore, the method has been further refined to incorporate oxidative cleavage for MC analogues containing modified Adda moieties such as ADMAdda and DMAdda, resulting in the formation of oxidation products analogous to MMPB [37].

Use of a single detection method presents risk. Non-specific biochemical methods are associated with matrix effects, are unable to determine toxin profiles, have cross-reactivity towards inactive metabolites and are linked to false positive detection [70,71,72]. Risks of false negatives and the absence of accurate toxicity equivalence factors for many cyanotoxin analogues [73] can affect the accuracy of targeted instrumental methods for assessing toxicity levels. Furthermore, impracticalities including sample throughput and turnaround time limitations are still associated with the potential for routine high-throughput monitoring using non-targeted mass spectrometry methods and potentially also MMPB analysis. Consequently, there are clear benefits to utilising multiple complementary detection methods to ensure toxicity is not under- or over-estimated, ideally incorporating a measure of both soluble and total MCs. 

Noting the priority for development of complementary cyanotoxin testing methods in complex matrices [6], before any testing methods can be implemented into monitoring programs, extensive validation of method performance is required. In addition, routine running of instrumental methods requires the application of a range of quality assurance and quality control procedures. These both require the use of matrix Reference Materials (RMs), so the availability of materials prepared from matrices of direct relevance to the test samples is important to laboratories wishing to implement and maintain cyanotoxin assays. Various RMs have been prepared for cyanotoxins in the past but focused on algal matrix. These include a certified reference material (CRM) for total MCs from the National Institute for Environmental Studies (NIES) in Japan which contained seven microcystin variants [74], as well as a multi-analyte feasibility study RM from NRC Canada containing seven MC analogues, anatoxins and cylindrospermopsins [75]. Multiple shellfish RMs have been developed, characterised and even certified in recent years for marine toxins [76,77,78,79,80,81,82,83,84,85,86,87,88,89], but to date no shellfish tissue RM exists for cyanotoxins. 

The need for such a RM prompted the culturing of toxic *Microcystis* and *Nodularia* with subsequent feeding studies in mussels in a controlled laboratory environment to assess the uptake and depuration of toxins within mussel tissues [26], with the resulting tissue materials utilized for RM preparation and characterization. The aim was to produce a shellfish tissue cyanotoxin RM which was fully characterised and provided proof of concept for future development of a fully certified shellfish RM containing a wide range of toxins from cyanobacterial origin. 

## 2. Results

### 2.1. Material Production and Initial Characterization

Cyanotoxin-contaminated mussel tissues were produced as detailed previously [26] and as described in the methods section. A culture of *Mycrocystis aeruginosa* utilized for shellfish feeding contained the microcystin (MC) variants (MC-LR, LW, LF, HilR, LY and desmethyl MC-LR (dmMC-LR, constituting [D-Asp^3^]MC-LR and/or [Dha^7^]MC-LR). A culture of *Nodularia spumigena* contained both NOD-R and smaller proportions of [seco-2/3]NOD (referred to previously as linear NOD or L-Nod) [26]. After mussel exposure, cyanobacteria-exposed mussel homogenates were mixed into one combined tissue for reference material preparation and homogenised further as per Section 4.3.

**Figure 2:**
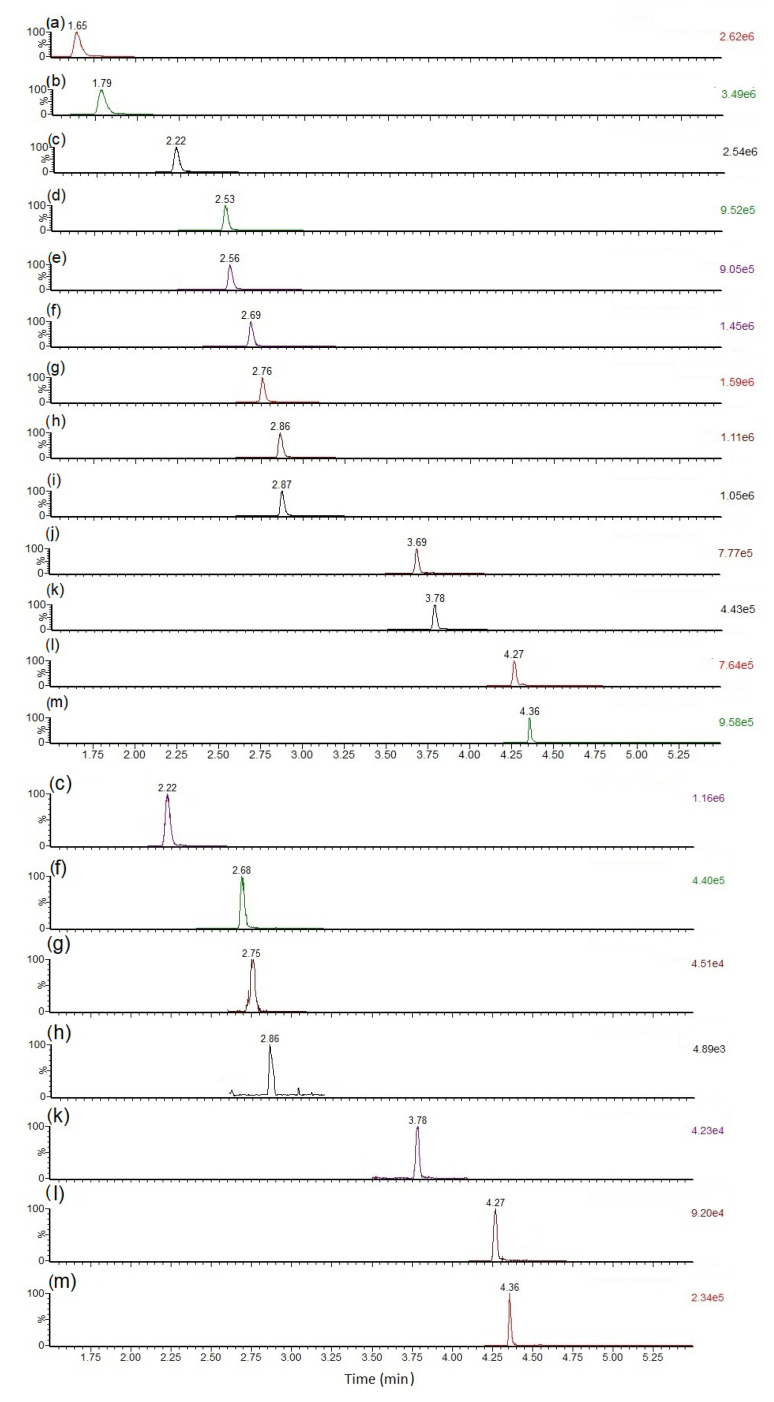
Total ion chromatograms summing SRM transitions (no mass spectral smoothing) for MCs and NOD in calibration standard (left) and mussel matrix RM (right), with peaks labelled as: (**a**) [D-Asp^3^]MC-RR (513 > 103; 135) (**b**) MC-RR (520 > 102.8; 1345 (**c**) NOD-R (825.5 > 135; 103) (**d**) MC-YR (1045.6 > 135; 127) (**e**) MC-HtyR (1059.6 > 135; 107) (**f**) MC-LR (995.6 > 135; 127) (**g**) dmMC-LR ([D-Asp^3^]MC-LR and/or [Dha^7^]MC-LR (981.5 > 135; 107)) (**h**) MC-HilR (1009.7 > 135; 127) (**i**) MC-WR (1068.6 > 134.9; 107) (**j**) MC-LA (910 > 135; 107) (**k**) MC-LY (1002.5 > 135; 107) (**l**) MC-LW (1025.5 > 135; 126.8) (**m**) MC-LF (986.5 > 135; 213).

Cyanotoxins initially identified in the targeted analysis of mussel tissue material were found to be primarily NOD-R and the MC congeners MC-LR, MC-LY, MC-LF and MC-LW, together with lower relative concentrations of MC-HilR and [D-Asp^3^]MC-LR and/or [Dha^7^]MC-LR, with the latter two sharing Selected Reaction Monitoring (SRM) transitions and retention times [90], so collectively referred to as dmMC-LR. Selected Reaction Monitoring (SRM) peaks generated following LC-MS/MS analysis are illustrated in Figure 2, showing the chromatographic peaks in a solvent-based calibration standard in comparison with those detected in the matrix RM. Retention times were found to be identical in the mussel sample extract in comparison to those in the methanolic standard as reported previously [90]. SRM transitions are summarized in Appendix A (Table A1). Preliminary quantitative results confirmed successful uptake of toxins into the whole combined toxic mussel tissue, with analysis showing approximate concentrations ranging from 20 µg/kg to 940 µg/kg wet weight. Concentrations determined here and throughout the manuscript were all calculated in terms of wet weight. Mussels from compartments B, C and F which were not exposed to toxic cyanobacteria were also shucked and tested, confirming the non-detection of any MCs or NODs.

### 2.2. Homogeneity

Table 1 shows the concentrations of cyanotoxins quantified in the homogeneity study samples subjected to LC-MS/MS analysis following extraction of duplicate aliquots of 14 samples across the entire production batch. Data generated under repeatability conditions clearly evidenced acceptable homogeneity of the RMs, given the low relative standard deviations (RSD %) for the majority of analytes and F-test values all less than F-critical. Appendix B shows the homogeneity control charts illustrating the distribution of individual toxin concentrations together with mean values and associated standard deviations.

### 2.3. Stability

**Figure 3:**
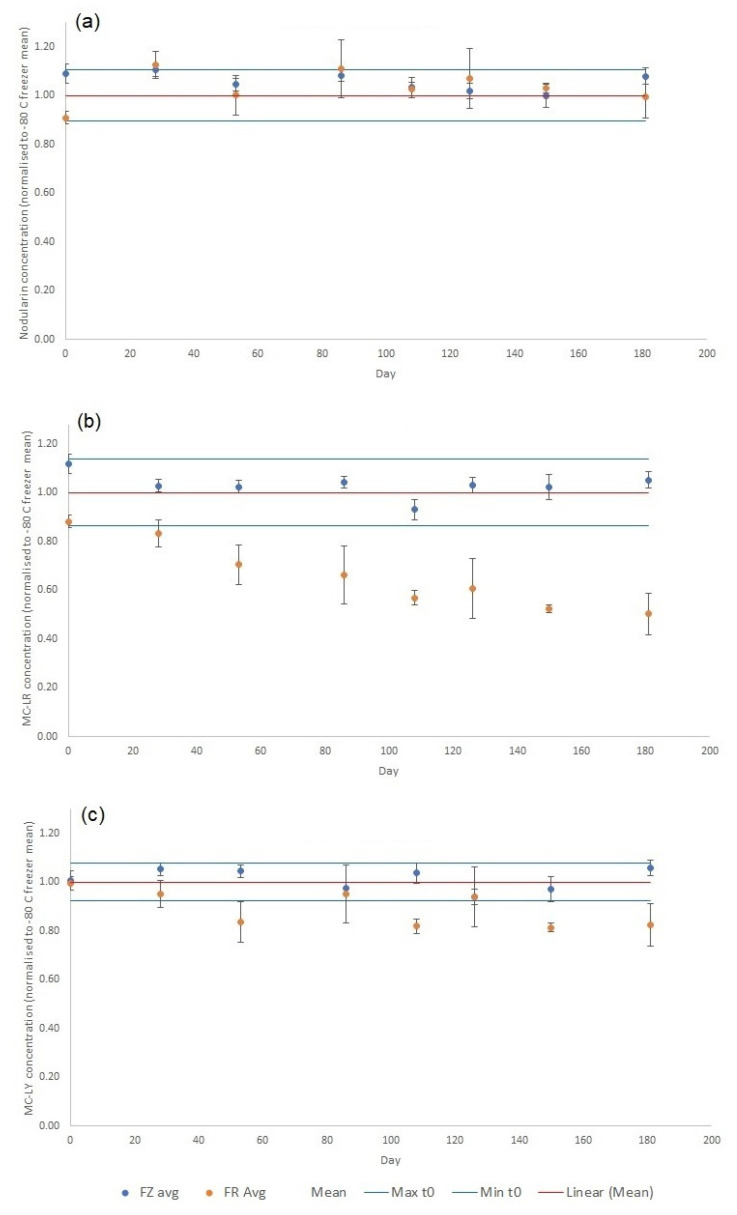

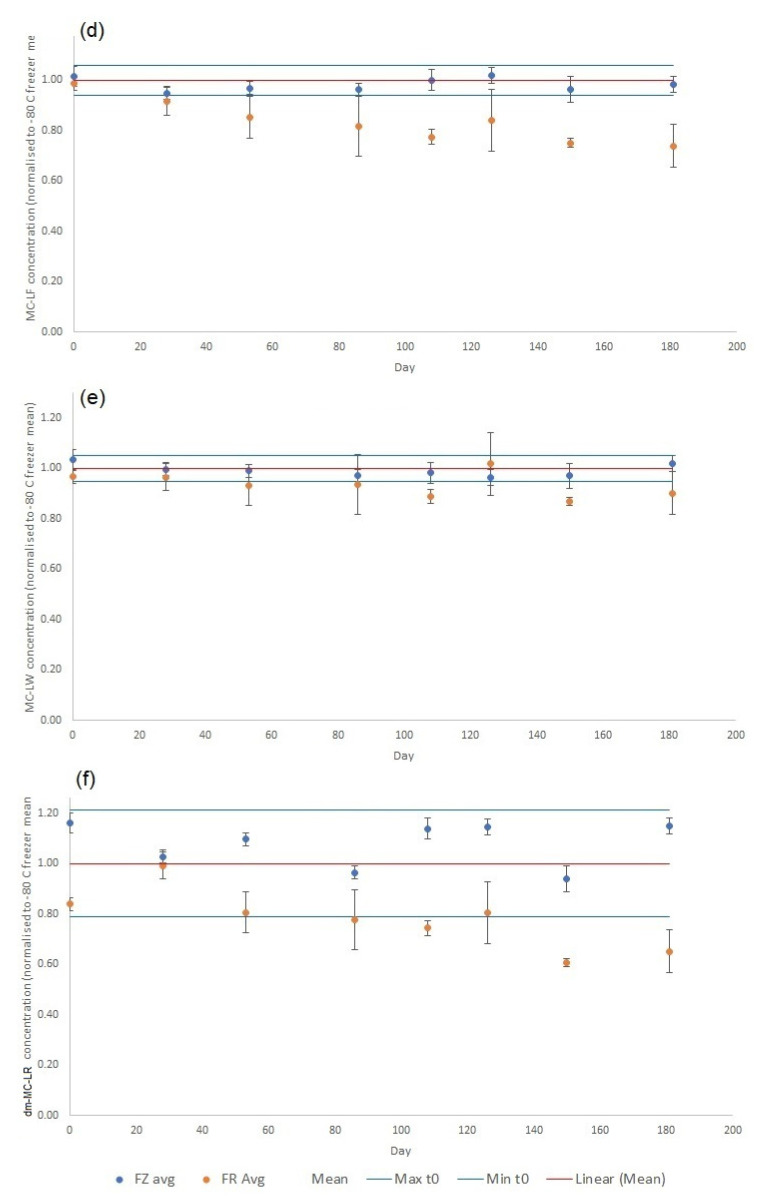
Cyanotoxin concentrations determined in crude extracts of wet RM tissues normalized to mean of time zero (*n* = 6) over 181-day stability experiment, showing effects of RM storage in freezer (FZ; −20 °C) and fridge (FR, +4 °C) for (**a**) NOD-R (**b**) MC-LR (**c**) MC-LY (**d**) MC-LF (**e**) MC-LW (**f**) dmMC-LR. Max = mean at time zero plus three times the standard deviation (*n* = 6); min = mean at time zero minus three times the standard deviation (*n* = 6).

Normalized quantitative stability data from the 181 day-long stability study are summarized in Figure 3 for each of the targeted analogues present in the RM (NOD-R, MC-LR, MC-LY, MC-LF, MC-LW and dmMC-LR). Acceptability limits were defined as three times the standard deviation of the mean concentrations determined at time zero (*n* = 6). The experimental assessment of toxin stability was assessed at both −20 °C and +4 °C, with stability results displayed here at both storage temperatures. The most significant instability under refrigeration was evidenced for MC-LR and dmMC-LR, where concentrations dropped to approximately 50% and 60% of mean initial concentrations, respectively after 181 days. Significant reduction outside of acceptability limits occurred for MC-LR and MC-LF as early as 28 days. The remaining MCs exhibited lower loss, with 74% to 90% remaining after 181 days at +4 °C. Interestingly, there was no evidence for concentration reductions for NOD-R in RMs stored in the fridge across the whole study period (Figure 3). For RMs stored in the freezer, stability was found to be within acceptability limits for all toxin analogues, with no evidence for concentrations dropping over the entire 181-day period. As such there was good evidence that storing cyanotoxin mussel tissue RMs in a freezer is suitable for maintaining the stability of analyte concentrations over the medium to long term.

### 2.4. Toxin Quantitation

Four different quantitative methods were applied to nine different tissue samples in order to compare quantitative results for freely-extractable soluble MCs + NODs and additionally to assess the presence of total MCs + NODs incorporating soluble toxins, soluble toxin-conjugates and protein-bound toxins. The samples incorporated triplicate mussel RMs, three different raw mussel tissue homogenates from each of the three different cyanobacterial exposure tanks (A, D and E) and a final three mussel tissue samples which were negative controls from tanks B, C and F. Each of the nine tissue samples were extracted and analysed blind using the quantitative LC-MS/MS method [90], the multihapten ELISA [42], the Adda ELISA using the refined approach of [65] and the GreenWater Labs in-house MMPB LC-MS/MS assay for total MCs + NODs [37]. Mean quantitative results for the triplicate RMs are summarised in Table 2**,** with full data for each individual material shown in Appendix C.

#### 2.4.1. LC-MS/MS

LC-MS/MS of the triplicate mussel RMs (RM1-3) returned values of 1096 ± 59 µg/kg and 1130 ± 51 µg/kg (wet weight) for the summed concentrations of MCs and NODs, respectively, resulting in a mean MCs + NODs combined of 2226 ± 33 µg/kg. Analysis confirmed the detection of the MC analogues MC-LR, MC-LY, MC-LF, MC-LW, dmMC-LR and MC-HilR as described earlier. In addition to cyclic NOD-R, the linear NOD analogue [seco-2,3]NOD was also detected and quantified, following confirmation of its presence by LC-HRMS, and included in the total NODs value. The three unprocessed exposed mussel tissue samples from tanks A, D and E were found to contain different total toxin concentrations, as expected given these were taken from separate tanks of mussels during the exposure process (Appendix C). Samples B, C and F, prepared from mussels unexposed to cyanobacteria contained no detectable presence of any targeted cyanotoxin analogues, as expected. Overall, this confirmed the consistent high proportion of NOD-R and the MC analogues MC-LR, MC-LF, MC-LW and MC-LY being the four highest MC analogues across all samples analysed.

#### 2.4.2. Multihapten ELISA

Total soluble MCs concentrations are also summarized in Table 2 following analysis of the nine samples using the multihapten ELISA [44]. In terms of qualitative identification of MC/NOD presence, the assay results agreed well with the LC-MS/MS. The three cyanotoxin-free tissues (B, C and F) showed no response using the assay, confirming the absence of any significant levels of MCs + NODs in the unexposed negative control tissues. For the triplicate RMs, the mean total concentration determined was 1424 ± 148 µg/kg wet weight (*n* = 3), on average 64% of the values quantified by LC-MS/MS for MCs + NODs combined, but 130% in comparison to MCs only by LC-MS/MS. For the three contaminated unprocessed samples, the ELISA was 27%, 39% and 66% of the LC-MS/MS result for samples A, D and E, respectively (Appendix C). 

#### 2.4.3. Adda ELISA

Qualitatively, the Adda ELISA also agreed well with the LC-MS/MS and multihapten ELISA data, with no toxin signal detected for any of the three unexposed toxin-free mussel samples B, C and F. In terms of quantitative concentrations for the remaining six samples, on average the concentrations returned by the ELISA were 94% of those by LC-MS/MS, although there was notable variability between samples. In particular the triplicate RMs showed values higher than LC-MS/MS, with the ELISA 160% of the LC-MS/MS on average, whereas sample A by ELISA was 44% of the LC-MS/MS and samples D and E 57% and 74%, respectively (Table 2). 

#### 2.4.4. 3-Methoxy-2-methyl-4-phenylbutyric Acid (MMPB) Analysis

The MMPB method was applied to both extracts of mussel tissues for determination of soluble toxins and to mussel tissues without extraction for determination of total toxin concentrations. The LC-MS/MS analysis of MMPB applied following oxidation of sample extracts from non-exposed samples showed no detectable presence of the oxidation products from MCs or NODs, therefore qualitatively agreeing with the results returned by both ELISAs and LC-MS/MS. MMPB analysis for freely extractable (soluble) MCs + NODs showed total concentrations on average 100 ± 15% of those total MCs + NODs concentrations quantified by LC-MS/MS (Table A2; Appendix C). 

**Figure 4:**
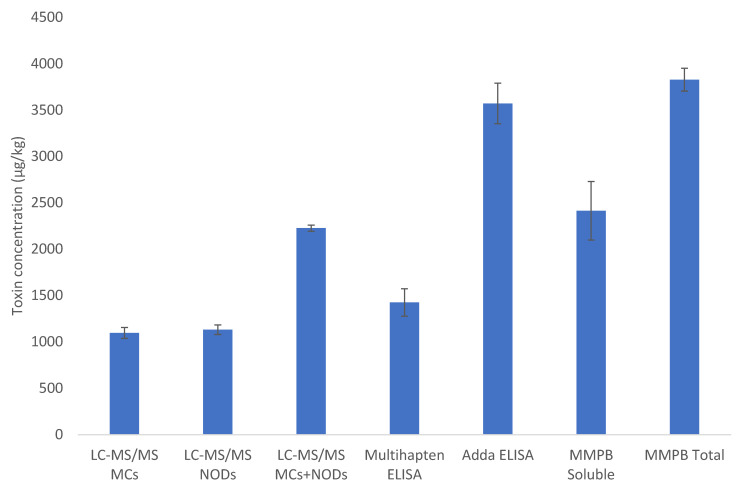
Bar chart showing mean toxin concentrations (µg/kg wet weight) quantified in RMs using the five quantitative methods: LC-MS/MS (showing results for total MCs only, total NODs only and total MCs + NODs combined), multihapten ELISA, Adda ELISA, MMPB for soluble MCs + NODs in extracts and MMPB for total MCs + NODs in RM tissues. Error bars show standard deviations associated with mean results (*n* = 3).

For the MMPB analysis of total MCs + NODs from whole tissue samples, again no toxins were detected in the unexposed mussel samples. For the RM tissues, total tissue concentrations were notably higher than the soluble MCs + NODs and LC-MS/MS with concentrations ranging from 136% to 215% (mean = 181 ± 27%) in comparison to the soluble MMPB results. Consequently, the comparison between soluble and total toxin data following MMPB analysis indicates that protein-bound or unextractable toxins can account for more than 50% of the total toxin content within the mussel tissues used for preparation of the cyanotoxin RM (Figure 4). 

**Table 2:** Summary of mean MCs + NODs concentrations (*n* = 3) with associated standard deviation (SD) and percent relative standard deviation (RSD %) and mean total toxin concentrations (µg/kg wet weight) in crude extracts of LRM following analysis by four techniques: LC-MS/MS, multihapten ELISA, Adda ELISA and MMPB for soluble and total toxins.

	Mean	SD	RSD %
NOD-R	938	43	4.5
[seco-2/3]-NOD	192	12	6.3
MC-LR	496	33	6.6
MC-LY	99	4.0	4.1
MC-LF	238	19	8.2
MC-LW	192	12	6.1
MC-HilR	15	4.0	26.7
dmMC-LR	56	5.6	9.9
Sum of all MCs by LC-MS/MS	1096	59	5.4
Sum of NODs by LC-MS/MS	1130	51	4.6
Sum of all MCs + NODs combined by LC-MS/MS	2226	33	1.5
Corrected concentration incorporating additional analogues determined by LC-HRMS	2393	35	1.5
Multihapten ELISA	1424	148	10.4
Adda ELISA	3571	219	6.1
MMPB—soluble MCs + NODs	2414	316	13.1
MMPB—total MCs + NODs	3828	123	3.2

### 2.5. Non-Target Toxin Analysis

Filtered methanolic extracts of the RM were further assessed by high resolution mass spectrometry (HRMS) using a data independent acquisition screening method. This relied on detection of characteristic microcystin product ions (e.g., *m/z* 135.0804, 213.0870, 375.1915) during sequential fragmentation of all precursor ions using 62 *m*/*z* selection windows. Subsequently, targeted MS/MS analyses were undertaken to obtain confirmatory data on any suspected targets. LC-HRMS/MS analysis confirmed the presence of all targeted analogues described by the LC-MS/MS approach above. Table 3 lists the targeted analogues in order of chromatographic peak area, as well as mass accuracy in relation to theoretical accurate mass. All positive identifications had product ion *m/z* within 5 ppm of theoretical values, and were all confirmed with MS/MS experiments, thereby providing excellent additional confirmation of the primary cyanotoxins present in the RM. The LC-HRMS analysis also confirmed the small relative proportion of [Dha^7^]MC-LR, which was not separated from [D-Asp^3^]MC-LR using the LC gradient used in targeted LC-MS/MS, and thereby provided additional evidence for the presence of both co-eluting dmMC-LR analogues, with a ratio of 1 to 0.14 between [D-Asp^3^]MC-LR and [Dha^7^]MC-LR (Table 3). Appendix D illustrates the LC-HRMS chromatograms obtained for those MCs and NODs measured by targeted LC-MS/MS.

**Figure 5:**
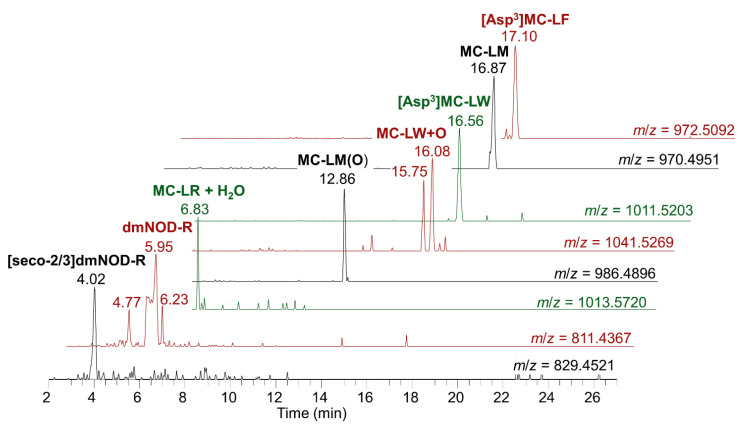
Mussel tissue RM chromatograms showing additional MC and NOD analogue peaks identified using LC-HRMS.

In addition to the nine targeted analogues confirmed by LC-HRMS, the analysis revealed the presence of multiple additional toxin analogues (Table 3), none of which were available as standards for additional confirmation. The two compounds resulting in the highest peak areas were [D-Asp^3^]MC-LW and [D-Asp^3^]MC-LF, present at 7.1% and 6.0% of the peak area of NOD-R, respectively. Other analogues included the methionine substituted MC-LM, together with oxidized variants of MC-LM and MC-LW and several desmethyl NOD-R isomers including [Dhb^5^]NOD, and at relative proportions of <0.5% NOD-R peak area, several putative MC-LR isomers. These minor toxin analogues detected by untargeted LC-HRMS/MS represent approximately 7.5% correction to the summed “MCs + NODs” concentrations as measured by targeted LC-MS/MS and presented in Table 2. This correction brings the LC-MS results in closer agreement with those from the Adda ELISA and MMPB, though would not be expected to account for contributions from bound MCs in the “total MCs + NODs” measurement by the MMPB method. Figure 5 shows the LC-HRMS chromatograms for the analogues detected only by LC-HRMS, listed in Table 3. Small molecule conjugates between the targeted MCs and thiols such as cysteine, glutathione and its degradation products that have been detected previously in high-level bloom samples were not detected in methanolic extract of the RM [51,52]. 

Subsequently, a second targeted LC-MS/MS method was set up to detect the minor cyanotoxin analogues detected by untargeted LC-HRMS, using the diagnostic *m/z* 135 product ion. The analogues incorporated were dmNOD (811.4 > 134.9), [seco-2/3]dmNOD (829.4 > 134.9), MC-LM (970.5 > 134.9), [D-Asp^3^]MC-LF (972.5 > 134.9), [D-Asp^3^]MC-LW (1011.5 > 134.9) and the two oxidized analogues of MC-LY and MC-LW (1018.5 > 134.9 and 1041.5 > 134.9, respectively) (Appendix A). Results showed SRM peaks indicative of the majority of these analytes, with the exception of MC-LM where no clear peaks were observed. Conversely, peaks of high intensity were seen particularly for dmNOD/ [Dhb^5^]NOD, where multiple peaks were detected (Appendix E), consistent with LC-HRMS results. 

### 2.6. Consensus Concentrations

**Figure A4:**
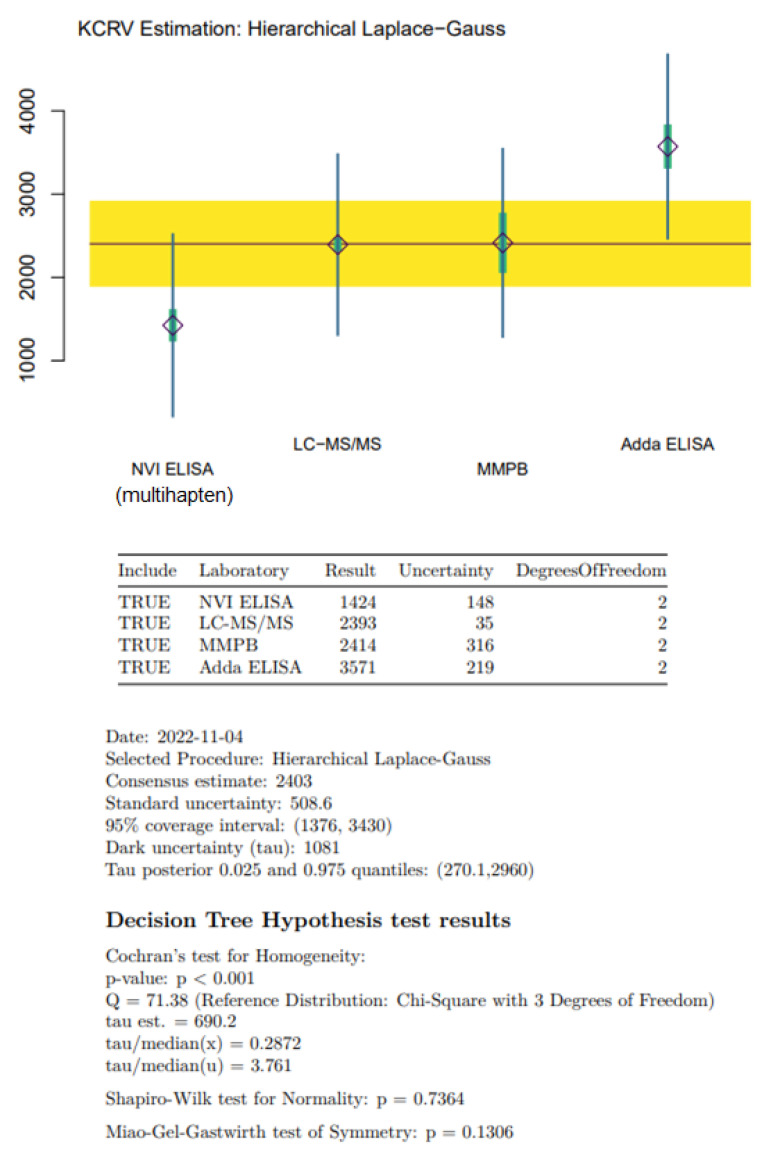
Summary of outputs generated following application of NIST Decision Tree and Hierarchical Laplace-Gauss Fit Model for consensus concentration assessment.

**Figure A5:**
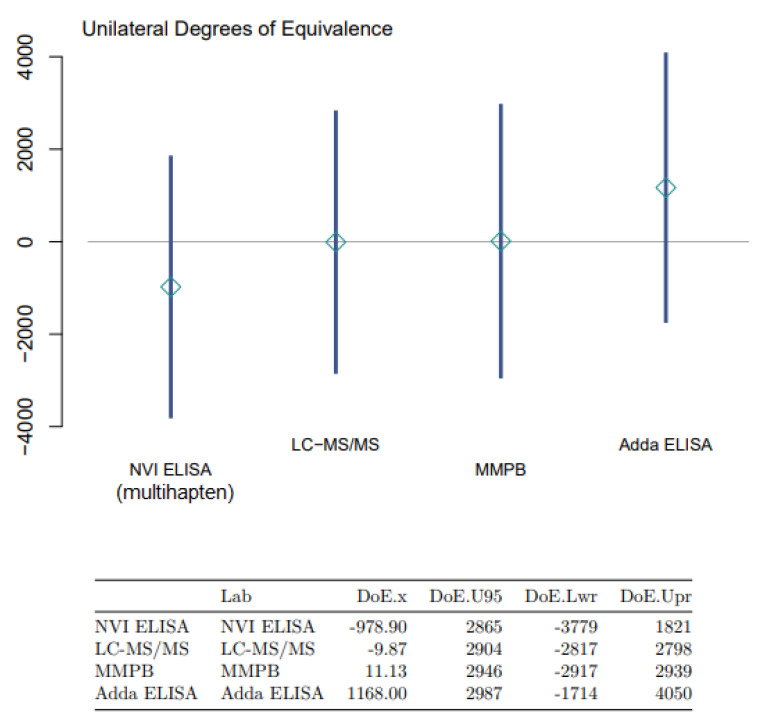
Statistical assessment of quantitative results between assays.

Concentrations of total soluble MCs + NODs determined using the four independent measurement methods were statistically assessed in order to generate a consensus value. The NIST Decision Tree (NDT) approach [91] based on [92] was utilized, by entering the measured values, associated standard uncertainties and measurement degrees of freedom (Table 2). Concentrations used were the multihapten ELISA, LC-MS/MS (adjusted for additional minor analogues), MMPB for soluble toxins and the Adda ELISA. The Cochran’s test [93] for data homogeneity demonstrated heterogeneous results (*p* < 0.001), so data homogeneity was not assumed. Symmetry and normality were both evidenced with application of the Miao-Gel-Gastwirth test of symmetry (*p* = 0.12) and the Shapiro–Wilk test of normality (*p* = 0.74), respectively [94,95]. Consequently, a Hierarchical Lapalace-Gauss fit model was used. The consensus estimate was 2425 µg/kg wet weight with an associated standard uncertainty of 575 µg/kg, with 95% coverage intervals of 1285 µg/kg and 3566 µg/kg. Raw output from the assessment is shown in Appendix F, Figure A4 and Appendix G, Figure A5.

## 3. Discussion

### 3.1. RM Preparation

Given increasing reports of cyanotoxins in edible aquatic organisms such as finfish and shellfish, e.g., [20,96,97] and subsequent impacts on human health, it is becoming more important to develop fit-for-purpose approaches for evaluating cyanotoxin presence in these complex biological matrices [34]. Accredited, validated routine testing methods require the application of strict quality assurance/control process, incorporating amongst other things, the analysis of positive control matrix materials. As such, this study targeted the production of a shellfish material containing both MCs and NODs, for use in routine analysis of samples harvested from shellfisheries impacted by potentially toxigenic cyanobacteria.

Given the practical challenges associated with collecting marine or estuarine shellfish naturally contaminated with freshwater cyanobacterial toxins, the laboratory-controlled exposure approach conducted through feeding mussels with toxigenic *Microcystis* and *Nodularia* resulted in mussel tissues containing a range of different toxins. LC-MS/MS quantitation confirmed the presence of the microcystin analogues MC-LR, MC-LY, MC-LF, MC-LW, dmMC-LR ([D-Asp^3^]MC-LR and/or [Dha^7^]MC-LR) and MC-HilR together with the NODs, NOD-R and [seco-2/3]NOD. LC-HRMS with a 30 min run time confirmed the presence of these analogues, as well as detecting both dmMC-LR analogues [D-Asp^3^]MC-LR and [Dha^7^]MC-LR which co-eluted under the implemented LC conditions. Toxins present at highest concentrations were the nodularins NOD-R and [seco-2/3]NOD (938 ± 43 µg/kg and 192 ± 12 µg/kg, respectively), along with the MCs MC-LR (496 ± 33 µg/kg), MC-LF (238 ± 19 µg/kg), MC-LW (192 ± 12 µg/kg) and MC-LY (99 ± 4.0 µg/kg), with all concentrations expressed in terms of wet weight. As such, the targeted toxin analogues present in this shellfish RM are those commonly encountered in tissue samples following cyanobacterial blooms around the world (e.g., [97]).

### 3.2. Method Comparisons

In terms of qualitative detection, the methods applied in this study all successfully distinguished between toxic and non-toxic mussel tissues. Such a screening approach would at least provide a useful first step in the assessment of food safety following a potential cyanotoxin exposure event. Statistical analysis demonstrated that method choice did have a significant effect on quantitative results generated, albeit on a low number of samples (Appendix G). This concurs with reports from various authors (e.g., [23,27]) who have reported that MC concentrations were significantly different for fish tissue samples when analyzing using different testing methods, with advantages and disadvantages associated with available methods [63]. ELISA methods, whilst quantitative, have previously been referred to as semi-quantitative, given the cross-reactivity with inactive MC metabolites and potential matrix effects [71,72]. Targeted and specific approaches using LC-MS/MS methods benefit from a high degree of specificity and sensitivity so are highly appropriate for MC quantitation [71,98]. The LC-MS/MS method applied in this study was fully validated in mussel matrix and demonstrated excellent method recovery as well as the lack of any significant matrix effects from the mussel tissue matrix [90]. However, the method relies upon primarily non-certified commercial reference standards for each analyte of interest, without which, and alongside the inability to detect protein-bound toxins and toxin metabolites, total MC concentrations can be underestimated. Such underestimation can be avoided through use of the MMPB method for quantitation of Adda-containing MCs, but analogue identification is lost [73]. Additionally, there is the potential, along with the Adda ELISA, for missing non Adda-containing analogues, unless refined to incorporate modified Adda moieties [37]. MMPB and ELISA methods typically compare well for confirming MC concentrations in water samples, with chromatographic methods carrying a risk of under-estimation [63]. However, further work is required to assess comparative performance in biological tissues, especially bivalve molluscs such as mussels.

In this study, two ELISA methods were used for quantitative assessment of MC/NOD concentrations in the mussel tissues. Different quantitative results returned by the two assays potentially relate to either differing assay functionality and/or differences in congener cross-reactivities [99]. Both ELISAs have acceptable method performance characteristics as determined through single-laboratory validation exercises, including mean recoveries of close to 100% and precision ≤15% [44,100], although notably both assessments focused on MC-LR only and were not conducted on mussel tissue matrix.

The multihapten ELISA [44] compared fairly well with the total MCs results determined by LC-MS/MS in the RMs, but under-estimated LC-MS/MS concentrations when including NODs into the total LC-MS/MS assessment (Table 2). These differences may be due to the assay being based on polyclonal antibodies raised against a mixture of different MC analogues, specifically MC-LA, MC-LF, MC-LW, MC-YR and MC-WR [44]. Consequently, the assay is specific to a range of MCs, with molar cross-reactivities of MC-LR (100%), MC-LY (46%), MC-LF (100%), MC-LW (93%), [Dha7]MC-LR (92%) [44]. On the other hand, the cross-reactivity for NOD-R was 58% to 72% in comparison with MC-LR, depending on the source of NOD-R [44]. This cross-reactivity can explain the fairly good correlation compared with the total MCs results determined by LC-MS/MS in the RMs and the slight under-estimation in total MCs + NODs in comparison to LC-MS/MS, especially considering the high level of NOD-R in the RM.

Conversely, the data returned using the Adda ELISA was higher on average than the total MCs + NODs combined. This assay, utilized for official method USEPA 546 [100], is an indirect competitive ELISA based on detection of the Adda epitope. QC checks for the validated method included thresholds for calibration correlation coefficients, replicate precision and spike recoveries, with all controls passing, although the sample matrix assessed was water, as opposed to tissues assessed in this study. The broad specificity of this assay may explain in part why it returned higher concentrations than the multihapten ELISA (Table 2). Notably, the Adda ELISA has been reported to show a mean 200% cross-reactivity for NOD-R, which is likely to contribute to the higher results from this assay [101]. Higher concentrations determined in comparison with LC-MS/MS may also infer that the Adda ELISA is incorporating additional minor analogues, potential degradation products or additional conjugated metabolites, which are not targeted by the chemical detection approach. It is also noted that untargeted LC-HRMS evidenced the presence of additional analogues which increased LC-MS/MS results by approximately 7.5%, with no conjugates, other Adda-containing degradation products or MCs with modified Adda moieties detected. As such, the comparison between Adda ELISA and LC-MS/MS at least agrees with reports that targeted chromatographic quantitation methods should return lower total MC concentrations in comparison to the more broadly specific biochemical methods [97,101,102]. 

Concentrations determined by MMPB analysis of soluble MCs + NODs in solvent extracts were found to compare closely with the total MCs + NODs results returned by LC-MS/MS, although there was less of an agreement between the MMPB and ELISA results, potentially providing further evidence for a lower cross-reactivity of the multihapten ELISA to NODs in comparison to MCs, assuming an equivalent response to all Adda-containing MC analogues (Table 3). 

Overall, however, the assessment provided an excellent multi-method assessment of freely-extractable cyanotoxins in the RM, giving greater confidence in the results obtained, highlighting some differences in ELISA performance, the benefits of using MMPB for quantifying soluble MCs + NODs, whilst profiles and total MCs + NODs values were confirmed using LC-MS/MS. 

### 3.3. Bound Toxins

Of all the methods employed, the MMPB LC-MS/MS analysis of oxidized tissue samples is uniquely able to measure bound MCs, even if they are not extractable by the sample preparation approach used for all other methods. Chromatographic quantitation methods such as LC-MS/MS will consequently determine lower toxin concentrations in comparison to the MMPB LC-MS/MS method after oxidative cleavage of Adda-containing MC/NOD congeners, given the ability of the latter to detect both protein bound and conjugated toxins [97,101,102]. The comparison of MMPB concentrations quantified in both solvent extracts and mussel tissue in this study demonstrated that a significant proportion of toxins present in shellfish were indeed bound to protein phosphatases and/or other biomolecules not soluble in the extraction solvent. Whilst these bound MCs may not be fully bioaccessible and therefore bioavailable following oral ingestion and digestion [20,60], the MC-protein phosphatase binding and/or the potential for cleavage of the covalent bonding may still result in the release of toxins from the contaminated seafood [23,68] given the reversible nature of thiol conjugation, although more information is required regarding deconjugation at low pH [53]. Whilst the MMPB method may need further refinement and standardization for tissue samples [23,37,63], the method has been utilized for certifying a water RM for microcystins in the past [74]. The method has usefully demonstrated the presence of bound toxins, which may add to the overall toxicity of contaminated shellfish following oral ingestion by humans, potentially increasing further following food processing [103,104,105]. 

Little data is available describing the range of proportions of protein-bound/conjugated cyanotoxins (masked toxins) in shellfish tissues, with the major focus to date being on bioaccumulation in finfish. In this study, the proportion of bound toxins ranged from 25% to 54% of the total toxin load (mean = 43 ± 9%) evidencing the significant levels of additional toxins within shellfish matrix in addition to the soluble toxins. It is noted that these bound/conjugated toxins have reached levels around 40% of the total in a relatively short period of time, given the tissues were contaminated through short-term exposure of mussels to toxic cyanobacteria in the laboratory, with these samples collected after just five days feeding [26]. Similarly, radiolabeling work using ^14^C-labelled MC-LR injections in salmon, demonstrated up to 60% of toxins to form unextractable covalent forms within five hours of administration, showing the rapid kinetics of the irreversible binding of toxins with protein phosphatases [56]. Within the literature, covalently bound MCs can account for up to 99% of the total MCs present in tissue samples, with application of the MMPB method evidencing the proportion of masked toxins to reach 85% in wild fish population tissues [22]. Other work has demonstrated ~75% of toxin burden to be associated with covalently bonded MCs in salmon livers and even 99.99% bound toxins in crab embryo tissues [55]. These higher proportions in comparison to the mussel RMs may relate either to the mechanisms of toxin accumulation in fish tissues and/or the length of time fish were exposed to toxigenic cyanobacteria in the environment during the causative bloom. It is also noted that non-Adda moiety containing MCs such as [DMAdda^5^] and [ADMAdda^5^] congeners will not oxidatively cleave to form the MMPB ions. No such analogues were detected in our mussel tissues through untargeted LC-HRMS screening, however. If such analogues were present, they could be detected via monitoring of either [DMAdda^5^] and [ADMAdda^5^] related cleavage products, specifically 2-methyl-3-oxo-4R-methyl-5S-methoxy-6-phenylhexanoic acid (MOMAPH) and 2R-methyl-3S-hydroxy-4-phenylbutanoic acid (MHPB), or alternatively through use of a PP2A which has the same cross reactivity between Adda and non-Adda toxins [37].

### 3.4. Potential as Mussel Reference Material

The mussel RM prepared was found to be homogenous across the entire preparation batch. Four independent methodologies subsequently facilitated the assignment of a consensus mean concentration for total MCs + NODs in the RM. In terms of stability, all MC and NOD analytes were found to be stable when tissues were stored frozen for up to 181 days. Conversely, under refrigerated conditions, there were significant losses of all MC analogues present in the mussel tissue over the 181-day storage period, with evidence for degradation of some analogues clear after just one month. The apparent lower stability of MC-LR and [D-Asp^3^]MC-LR/[Dha^7^]MC-LR indicates that the analogues with arginine (R) at position 4 in the molecule are potentially more prone to degradation than the other analogues assessed in this material, given all MC analogues in our material were leucine (L) substituted at position 2, with others including position 4-substituted tyrosine (MC-LY), phenylalanine (MC-LF) and tryptophan (MC-LW) substituents. Conversely, other reports have evidenced lower stability in water samples for analogues incorporating tryptophan such as MC-LW and MC-WR (Dinh et al., 2020). Unfortunately, the microcystin profile generated in the tissue did not contain any analogues with arginine at position 4, thereby preventing the assessment of other commonly occurring analogue types such as MC-RR, MC-YR and [D-Asp^3^]MC-RR. Given that tissues were not sterilized, microbe-mediated degradation was expected, along with enzyme-related changes. Interestingly, NOD-R concentrations remained stable in refrigerated tissues for the whole study period, potentially relating to the absence of a reactive Mdha^7^ group in NOD (Figure 1), which could suggest that abiotic changes are dominant in the fridge-stored tissues for the MC analogues. Further work would be required to confirm this using sterilized tissue samples. With evidence for acceptable stability of all MCs and NOD-R in tissues stored in the freezer for six months, the results demonstrated that these RMs should be stored frozen for the long term, in order to prevent degradation of toxins (e.g., [106]). Whilst future work could assess stability beyond six months, this study does demonstrate a proof of concept for cyanotoxin stability in shellfish tissue materials when samples are stored correctly, without any apparent need for additional stabilization techniques as per (e.g., [77,78,79,86,87].

Previous work has highlighted the urgent need for standardizing cyanotoxin extraction methods and related testing methods [69,107] and to report quantitative results alongside matrix QC samples associated with certified or at least well characterised toxin concentrations in order to compare results generated between different methods [23]. The availability of a shellfish reference material for cyanotoxins addresses in part the need for generating data on the occurrence of cyanobacterial toxins in seafood matrices, other than fish [23]. As such, it is critical that well characterised materials ultimately become available to testing laboratories, in order to help facilitate more accurate and reproducible cyanotoxin testing in biological tissues. Additionally, certified or even well-characterised matrix materials will aid the validation of testing methods, particularly those incorporating the detection and quantitation of protein-bound toxins, noting that recovery spiking experiments will only help determine the extraction efficiency for unbound toxins [34]. Consequently it will be important to characterize and ultimately certify both the soluble and bound toxin fractions within any such tissue. 

Following on from this proof-of-concept investigation, in order to fully develop the capabilities for production of a fully certified cyanotoxin reference material, further work is recommended. Enhanced stability assessments incorporating (a) longer assessment times (b) position 4 arginine substituted analogues (c) comparative use of stabilization techniques [76] such as autoclaving [89], freeze-drying [78,86]), use of antioxidant and antibiotic additives [77,89], high-pressure processing [88] and gamma irradiation [79,87]. The development of additional certified calibration solutions along with isotopically labelled toxins facilitating isotope-dilution mass spectrometric determination could also be used to further improve method accuracy, precision and traceability of soluble toxin measurement. A suitable negative control tissue would also be recommended to run alongside the cyanotoxin RM, with recent non-targeted analysis on the NRC FDMT1 material revealing the absence of MCs, making this a potentially useful control RM [108]. Further methodological advancements incorporating de-conjugation approaches following [53] alongside current MMPB methods would also be useful to develop reliable approaches for measuring bound toxin concentrations. There would also be advantages to incorporate a larger number of cyanotoxins, including not only additional microcystin analogues, but also other toxin families such as cylindrospermopsin, anatoxins and saxitoxins, ultimately providing a unique yet important seafood matrix reference material for future validation and quality control procedures. Given the significant presence of bound toxins in the mussel tissues assessed in this study, there is also an important requirement to determine whether the bioavailability of this fraction is likely to cause equivalent toxicity to humans following consumption of contaminated products.

## 4. Materials and Methods

### 4.1. Chemicals and Standards

For shellfish feeding, Shellfish diet 1800 (approximately 7.4 × 10^11^ cells/mL) was purchased from ReedMariculture Inc. (Campbell, CA, USA), and dilutions were made in water/seawater (10:0.86, *v*/*v*). For LC-MS/MS analysis and toxin quantitation, analytical grade chemicals and HPLC-grade solvents were used throughout the study. Mobile phases were prepared from LC-MS-grade acetonitrile (Fisher Optima, ThermoFisher, Hemel, Hempstead, UK) and water used for LC-MS was obtained in-house. Toxin standards used for preparation of calibration solutions (MC-RR, MC-LA, MC-LY, MC-LF, MC-LW, MC-YR, MC-WR, MC-HilR, MC-HtyR, MC-LR, [D-Asp^3^]MC-LR and NOD-R) were obtained from Enzo Life Sciences, Exeter, UK (≥95% purity). A certified standard of [Dha^7^]MC-LR (~99.6% purity) was obtained from Biotoxin Metrology, National Research Council Canada (NRCC; Halifax, NS, Canada), but this was not incorporated into the final mixed calibrant solutions given the lack of chromatographic separation from [D-Asp^3^]MC-LR. A NRCC CRM for MC-LR (~97.5% purity) was also used for calibration of ELISA and MMPB methods. Reference standards received as solid films were dissolved in water:methanol (1:1 *v*/*v*), to form stock solutions. A mixed stock solution was subsequently prepared by combining aliquots of each stock, followed by further dilutions in solvent to create seven-level suite of working calibration standards between 0.33 ng/mL to 327 ng/mL per toxin. 

### 4.2. Culturing of Cyanobacteria and Shellfish Feeding

**Figure 6:**
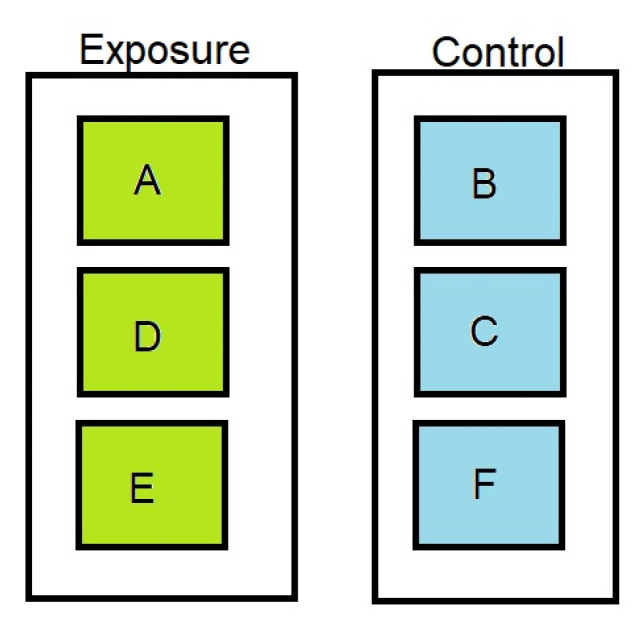
Schematic illustration of exposure and control tanks showing three sub-compartments for each exposure regime, based on [26]. A, D and E contained mussels exposed to cyanobacteria, whilst B, C and F were control tanks with no toxin containing cyanobacteria exposure.

Cyanobacterial culturing and shellfish feeding was conducted as detailed in [24]. In brief, two cyanobacterial cultures were grown in modified BG-11 medium with 75 g/L sodium nitrate: *Nodularia spumigena* KAC 66 (Kalmar Algae Collection, Kalmar, Sweden) and *Microcystis aeruginosa* PCC 7813 (Pasteur Culture Collection of Cyanobacteria, Paris, France). *N. spumigena* media was supplemented with 20% (*w*/*v*) Instant Ocean artificial seawater (Aquarium Systems Inc., Sarrebourg, France). Cultures were maintained at 20–23 °C, under continuous illumination (10–15 µmol/m^2^/s) and sparged with sterile air at 2.3 L/min until four days before harvesting. Cells were collected and maintained in 25 L carboys at 17 ± 1 °C with mild aeration and a light cycle of 17 h illumination (24 µmol/m^2^/s) and 7 h darkness for four weeks prior to shellfish feeding. Live mussels *Mytilus edulis* (sex undetermined) were obtained from the Shetland Islands (N Scotland, UK), acclimatized to laboratory tank conditions for a week and cleaned of barnacles and other debris prior to the experiment. Two seawater tanks (each 300 L, 122 cm long, 102 cm wide, 77 cm high and containing approximately 150 L seawater) equipped with ultraviolet sterilizers (class 1 IP64, twin UV 24 W, 240 V, 50 Hz, Tropical Marine Centre, Rickmansworth, Greater London, UK) were maintained at 16 ± 1 °C. One of the tanks was used for the exposure of 420 *M. edulis* to *M. aeruginosa* (3.9 × 10^6^ cells/L final concentration) and *N. spumigena* (3.1 × 10^6^ cells/L final concentration) for a total period of three days of daily feeding. Mussels were separated into three separate sub-compartments (A, D and E). Live cyanobacterial cells were homogenised and fed to live mussels. The second control tank contained 420 mussels separated into three sub-compartments B, C and F and fed shellfish diet only (Figure 6). After checking toxin concentrations within the tissues of a sub-sample, the exposure was terminated and the mussels removed for processing.

### 4.3. Reference Material Preparation

Just over 2.0 kg of toxin-containing mussel tissue from the exposed tank sub-compartments A, D and E was available for processing after the exposure study had completed and a previous study on MC/NOD uptake in mussels was completed [26]. Mussels were shucked to remove shells and whole tissues incorporating all the visceral mass and edible muscle combined were homogenised thoroughly in batches using high speed Waring industrial blenders. Each batch of homogenised tissue was combined into a large polypropylene container, with approximately 450 mL deionized water added to further aid homogenization, resulting in an overall water content of ~80%. Re-homogenization was conducted using a high-speed hand blender and an aliquot of tissue was taken for analysis to confirm the presence of a suitable number of cyanotoxin analytes at appropriate concentration levels. After testing, a final homogenization step was conducted and a magnetic stirrer added to the vessel, enabling continuous stirring during reference material (RM) aliquoting. Prior to dispensing the samples, 7.0 mL polypropylene Bijou vials were labelled with unique LRM identification numbers. Aliquots (4.5 g) were weighed into the tubes, to provide enough material for two 2.0 g sub-samples to be taken from each vial. As soon as each vial was dispensed, a second operator capped the tube. A total of 520 vials of RM were dispensed and capped, before placing all upright in a −20 °C freezer in order to freeze the contents in the bottom of the vials. After 24 h, the vials were transferred to long-term storage at −80 °C. 

### 4.4. Targeted Toxin Analysis

Vials of mussels were opened carefully and 2.00 ± 0.01 g sub-samples were weighed accurately into 50 mL polypropylene centrifuge tubes. Extraction and analysis by LC-MS/MS was conducted using the validated and ISO17025:2005 accredited method of [90]. Briefly, each homogenised tissue was extracted with a single dispersive extraction using 8.0 mL of methanol:water (80:20, *v*/*v*) and a 2 min vortex mixing time prior to centrifugation (4500 g; 10 min) and filtration of the resulting supernatant (0.2 µm syringe filter). Targeted LC-MS/MS analysis of cyanotoxins was conducted as detailed in [90]. A Waters (Manchester, UK) Acquity UHPLC system coupled to a Waters (Manchester, UK) Xevo TQ triple quadrupole mass spectrometer (MS/MS) was used with a 1.7 µm, 2.1 × 50 mm Waters Acquity UPLC BEH C18 column in conjunction with a Waters BEH C18 guard cartridge. The column was held at +60 °C, and a 5 µL injection volume utilized, together with mobile phase flow rate of 0.6 mL/min. Mobile phase A1 consisted of water containing 0.025% (*v*/*v*) of formic acid, mobile phase B1 comprised acetonitrile (MeCN) with 0.025% (*v*/*v*) formic acid. The UHPLC gradient started at 98% A1, dropping to 75% A1 at 0.5 min holding until 1.5 min, dropping further to 60% A1 at 3.0 min, decreasing further to 50% A1 at 4 min, before a sharp drop to 5% A1 at 4.1 min, holding until 4.5 min before increasing back to 98% A1 for column equilibration at 5 min for a further 0.5 min. The MS/MS source parameters were exactly as specified in [90], with 150 °C source temperature, 600 °C desolvation temperature, 600 L/h desolvation gas flow, 0.15 mL/min collision gas flow and capillary voltage at 1.0 kV. Quantitation of MCs was performed against external calibration standards with results calculated in terms of µg/kg wet weight of shellfish tissue. Throughout each LC-MS/MS sequence used for characterization, homogeneity and stability testing, quality control (QC) measures were applied following ISO17025-accredited protocols. This included the assessment of instrument blanks, method sensitivity, calibration linearity, positive control response, method procedural blanks and method precision. All QC checks passed stipulated thresholds. For minor toxin analogue analysis, the same chromatographic conditions as above were used. However, LC effluent was directed into the ESI source of a Waters Xevo TQ-S triple quadrupole mass spectrometer operating in SRM scan mode using positive ionization. SRM transitions are shown in the chromatograms of Figure 2 and were obtained using a collision energy of 70 eV as per [90]. Appendix A tabulates all SRM transitions. Full method performance characteristics for the method are detailed in [90]. Limits of Reporting (LOR) range from 0.3 to 1.5 µg/kg wet weight per toxin in mussel tissue, with the majority of calibration regressions >0.99 over a linear range reaching the equivalent of 2.5 mg/kg wet weight. No significant matrix effects were evident, so all calibrations were subsequently prepared in solvent rather than mussel extract matrix.

### 4.5. Toxin Profile and Homogeneity Testing

Fourteen RM samples were chosen for homogeneity testing. These were selected immediately after aliquoting and initial freezing, to provide a representative cross section of samples aliquoted over the entire production batch. In addition to the first (vial 1) and last (vial 520) aliquots dispensed, remaining test samples were randomly chosen across the production batch. Prior to homogeneity testing, samples were allowed to slowly thaw to room temperature, before the contents of each sample were mixed and two separate 2.00 ± 0.01 aliquots weighed out from each vial into 50 mL polypropylene centrifuge tubes, giving a total of 28 samples for extraction. All samples were extracted in MeOH:water (80:20 *v*/*v*) and filtered prior to LC-MS/MS analysis. 

### 4.6. Stability Testing

Material stability was assessed over 181 days using a reverse-isochronous experimental design. Triplicate RM vials were subjected to storage under both frozen (−20 °C) and refrigerated (+4 °C) conditions, incorporating eight time points (specifically 0, 28, 53, 86, 108, 126, 150 and 181 days). The triplicate samples were stored at −80 °C for variable periods of time before being placing in storage at the two elevated temperature conditions until the end of the study. At the end time point, all samples were removed from storage, allowed to equilibrate to room temperature before being extracted, filtered and analysed by LC-MS/MS. During analysis, triplicates were spread across the entire instrumental sequence, to account for any instrumental drift if present. Quantitative results determined at t = 0 were used to calculate mean concentrations, with three-times the standard deviation of the mean used to assigned acceptability limits for toxin concentrations at later time points.

### 4.7. Untargeted Toxin Analysis

Sample extraction was carried out as in Section 4.4. LC-HRMS analyses were performed on an Agilent 1200 LC system (Agilent, Santa Clara, CA, USA) coupled to a Q Exactive HF Orbitrap mass spectrometer with a HESI-II heated electrospray ionization interface (ThermoFisher Scientific, Waltham, MA, USA). Liquid chromatography parameters included a 5 µL injection on a SymmetryShield 3.5 µm C18 column (100 × 2.1 mm; Waters, Milford, MA, USA) held at 40 °C with mobile phases A and B of H_2_O and CH_3_CN, respectively, both of which contained 0.1% *v*/*v* formic acid. The elution gradient (0.3 mL/min) included a linear increase from 20–100% B over 21 min, a hold at 100% B (6 min), a decrease to 20% B over 0.1 min and equilibration at 20% B for 2.9 min. 

LC-HRMS source conditions included a capillary temperature of 350 °C, sheath and auxiliary gas flow rates of 25 and 8 units, respectively a spray voltage of +3.7 kV and an S-Lens RF level of 100. Comprehensive HRMS/MS data were first acquired using a combined full scan (FS) and data independent acquisitions (DIA) scan mode. All HRMS/MS data were processed manually in Thermo Xcalibu 4.1 software.

Full scan data were collected from *m/z* 500–1200 using the 30,000 resolution setting, an AGC target of 1 × 10^6^ and a max IT of 120 ms and MS/MS data was collected using the 15,000 resolution setting, an AGC target of 2 × 10^5^ and a stepped collision energy of 30, 60 and 80 V. For DIA, the max C-trap ion trapping time (max IT) was set to ‘auto’ and 62 *m/z* wide precursor isolation windows centered at *m/z* 530, 590, 650, 710, 770, 830, 890, 950, 1010, 1070, 1130, 1190, 1250, 1310, and 1370 were used. 

Targeted MS/MS spectra of suspected MCs were then acquired using the Parallel Reaction Monitoring scan mode with a using a resolution setting of 15,000, AGC target of 5 × 10^5^ and, a Maximum IT fill time of 3 sec and an isolation window of 0.5 *m/z*. Stepped CE for MCs containing one arginine residue were 60, 65 and 70 V and those for MCs containing no arginine residues were 25, 35 and 40 V.

### 4.8. ELISA

#### 4.8.1. Multihapten ELISA

Mussel tissues were extracted by adding either 8.0 mL or 10.0 mL MeOH:water (80:20 *v*/*v*) to 2.0 g tissue homogenate, vortex mixing and then centrifuging for 10 min (2250 g; 4 °C). The concentration of MCs in each shellfish extract supernatant was determined by indirect competitive ELISA (NVI, Oslo, Norway) as described by Samdal et al. [42]. The assay was optimized, as reported previously, with only minor adjustments to 0.5 µg/mL of the plate-coating antigen, 1:7000 of the antiserum 80289-5b, and 1:12,000 of the donkey antisheep IgG (H + L)−horseradish peroxidase conjugate (antisheep−HRP from Agrisera antibodies (Vännäs, Sweden)). These concentrations were determined by checkerboard titrations followed by optimization of the standard curve. The MC-LR standard (NRC CRM-MC-LR) in methanol (500 ng/mL) was diluted in Phosphate Buffered Saline with Tween (PBST) to give a methanol concentration of 10%, and then in a threefold dilution series in sample buffer resulting in standard concentrations of 50, 16.7, 5.56, 0.62, 0.20, 0.069, 0.023, 0.0076, and 0.0025 ng/mL. Serial dilutions of standards and samples were analyzed in duplicate on the plate. All incubations were performed at ~20 °C. Absorbances were measured at 450 nm using a SpectraMax i3x plate reader (Molecular Devices, Sunnyvale, CA, USA). Assay standard curves were calculated using 4-parameter logistic treatment of the data using SoftMax Pro version 6.5.1. (Molecular Devices, Sunnyvale, CA, USA). The assay working range was defined as the linear region at 20–80% of maximum absorbance (A_max_). The CYNTX RMs were quantitated at 400 and 800 times dilution to remove potential matrix effects, as evidenced by negative control shellfish samples. 

#### 4.8.2. Adda ELISA

Tissues were transferred to glass vials as 100 mg subsets (in duplicate) and extracted using 4 mL MeOH:water (75:25 *v*/*v*) in 100 mM acetic acid via bath sonication (20 min). Samples were cooled (4 °C) followed by centrifugation (1500 g; 15 min). Supernatants were retained and pellets vortex rinsed with extractant followed by centrifugation. The supernatants were pooled, mixed, and used for both the Adda ELISA and subsequent MMPB oxidation. One of each duplicate ‘Soluble MCs + NODs’ extract (50 µL corresponding to 1 mg tissue) were evaporated to dryness (60 °C, N_2_) and reconstituted in 1 mL phosphate buffer (10 mM; pH 7) for analysis. Further dilutions were conducted as necessary to achieve data within range of the calibration curve (0.15–5.0 ng/mL of NRC-CRM-MC-LR). A MCs + NODs Adda ELISA (Eurofins-Abraxis, Warminster, Pennsylvania, USA) [109] was utilized with the protocol refined and QC requirements as previously described [101]. This included the preparation of a standard curve from dilutions of a certified MC-LR standard over a range between 0.15 ng/mL and 4.00 ng/mL, also incorporating fortified samples at 1.0 ng/mL. The assay is sensitive down to a quantification limit of 150 µg/kg wet weight for MCs + NODs as determined from dilution factors (1000-fold) and kit sensitivity (0.15 ng/mL). 

### 4.9. MMPB for Total MCs

#### 4.9.1. Sample Preparation

Samples for the MMPB method were further homogenized by transferring 0.5 g of material to 7 mL vials containing ceramic beads (2.8 mm). Each subsample received 10 mM phosphate buffer (pH = 7) to achieve sample concentrations of 100 mg/mL. An Omni Bead Ruptor was utilized to homogenize samples (6 m/s; 15 s; 2×; dwell 30 s). Aliquots (0.1 g corresponding to 10 mg) were dispensed via Pasteur pipette into glass vials for oxidations in duplicate. In addition to whole tissues, tissue extracts (Section 4.8.2) were oxidized as 500 µL aliquots (corresponding to 10 mg tissue in acidified MeOH) for freely extractable soluble MCs + NODs. All subsets were spiked with internal standard (*d_3_*-MMPB). Tissue was oxidized with 2.5 mL of oxidant while extracts were oxidized with 1 mL oxidant, which was composed of 0.2 M K_2_CO_3_, 0.1 M KMnO_4_ and 0.1 M NaIO_4_. Oxidation was stopped by the addition of sodium bisulfite (40% wt%). Aliquots were cleaned using solid phase extraction (SPE). Preconditioned Strata X Polymeric SPE cartridges (200 mg for tissues, 100 mg for extracts) were loaded with sample, rinsed 3x with deionized water, and eluted with acetonitrile:water (90:10 *v/v*). Elutions were evaporated to dryness (60 °C, N_2_), reconstituted in MeOH:water (5:95 *v/v*) (sample concentration of 10 mg/mL), filtered (0.2 µm), and analyzed. QC measures incorporated into the testing batch included application of spiked recovery checks and internal standard returns, together with method procedural blanks. All controls fell within method-specified control limits.

#### 4.9.2. LC-MS/MS Analysis for MMPB (Adda MCs + NODs)

The [M-H]^−^ ion of MMPB (*m/z* 207) was fragmented and the product ion *m/z* 131 was monitored. The internal standard *d_3_*-MMPB (*m/z* 210 > 131) was used in conjunction with MMPB response for calibration. A matrix matched calibration curve (using a non-exposed toxin-free mussel control sample) was generated for quantification of total Adda MCs + NODs (0–25,000 ng/g of oxidized MC-LR) and a calibration curve generated in water was used for quantification of soluble MCs + NODs (0–500 ng/mL of oxidized MC-LR). A certified reference standard of MC-LR (NRC CRM -MC-LR) was used for all calibrations.

### 4.10. Data Analysis

Statistics used for the assessment of method equivalence were performed using RStudio (version 1.3. 1056). For the repeated means ANOVA (‘rstatix’package) and Tukey’s pairwise post hoc (‘eemeans’ package) analysis data was log transformed. The NIST Decision Tree was used to provide a recommendation on how to combine the independent measurement results obtained from the ELISAs, MMPB and LC-MS/MS analyses [91,92], with a Hierarchical Laplace-Gauss Fit Model applied for determining the consensus estimate and associated standard uncertainty.

## Figures and Tables

**Table 1:** Mean toxin concentrations (µg/kg wet weight) in mussel tissue reference material quantified following homogeneity tests (*n* = 14; duplicate samples) together with associated standard deviations (SD), percentage relative standard deviations (RSD %) and F-test values (F-critical = 2.53).

	NOD-R	MC-LR	MC-LY	MC-LF	MC-LW	dmMC-LR	MC-HilR
Mean concentration	926	502	89.3	254	184	68.0	21.1
SD (*n* = 14)	27.1	17.4	2.76	8.18	5.52	3.92	2.78
RSD %	2.93	3.47	3.09	3.22	3.00	5.77	13.16
F-calc	0.54	0.47	0.67	0.52	0.42	0.50	0.77

**Table 3:** Microcystin (MC) and nodularin (NOD) analogues detected in mussel matrix RM using LC-HRMS showing compounds detected by targeted LC-MS/MS and additional putative MCs detected by LC-HRMS, associated retention times (R.T), determined pseudomolecular ions in positive electrospray mode [M + H]^+^, the theoretical mass and consequent mass error (ppm), together with percentage peak area relative to NOD-R.

	Analogue ^a^	R.T. (min)	[M + H]^+^	Theoretical *m*/*z*	Mass Error (ppm)	Peak Area (%) Relative to NOD-R
Targeted LC-MS/MS	NOD-R	6.25	825.4497	825.4505	−0.96	100
	MC-LR	7.18	995.5561	995.5560	0.10	48
	MC-LF	17.77	986.5219	986.5233	−1.4	40
	MC-LW	17.18	1025.5325	1025.5342	−1.7	37
	MC-LY	15.3	1002.5176	1002.5183	−0.70	12
	[D-Asp^3^]MC-LR	6.90	981.5413	981.5404	0.92	3.6
	[seco-2/3]NOD	4.35	843.4642	843.4611	3.7	3.2
	[Dha^7^]MC-LR	7.17	981.5414	981.5404	1.0	0.51
	MC-HilR	7.43	1009.5729	1009.5717	1.2	0.43
LC-HRMS	[D-Asp^3^]MC-LW	16.60	1011.5170	1011.5186	−1.6	7.1
	[D-Asp^3^]MC-LF	17.12	972.5076	972.5077	−0.10	6.0
	MC-LM(O)	12.89	986.4910	986.4903	0.71	2.2
	MC-LW(O)	16.09	1041.5284	1041.5292	−0.73	2.0
	dmNOD-R	5.93	811.4353	811.4349	0.46	1.9
	MC-LM	16.87	970.4966	970.4954	1.2	1.8
	MC-LW(O)	15.75	1041.5299	1041.5292	0.67	1.1
	dmNOD-R	5.60	811.4355	811.4349	0.69	1.0
	dmNOD-R	4.79	811.4361	811.4349	1.5	0.46
	[seco-2/3]dmNOD	4.02	829.44822	829.4454	3.4	0.39
	[Dhb^5^]NOD	6.25	811.4350	811.4349	0.099	0.30
	MC-LR + H_2_O	6.85	1013.5667	1013.5666	0.099	0.27
	MC-LR isomer	6.68	995.5570	995.5560	0.95	0.16
	MC-LR + H_2_O	7.12	1013.5678	1013.5666	1.1	0.048
	NOD isomer	6.69	825.4524	825.4505	2.3	0.047

^a^ Analogue name prefix “dm” denotes desmethylation at an unspecified position. Analogue suffix “(O)” indicates oxygen addition at an un-specified position to the amnio acid immediately preceding it.

**Figure A1:**
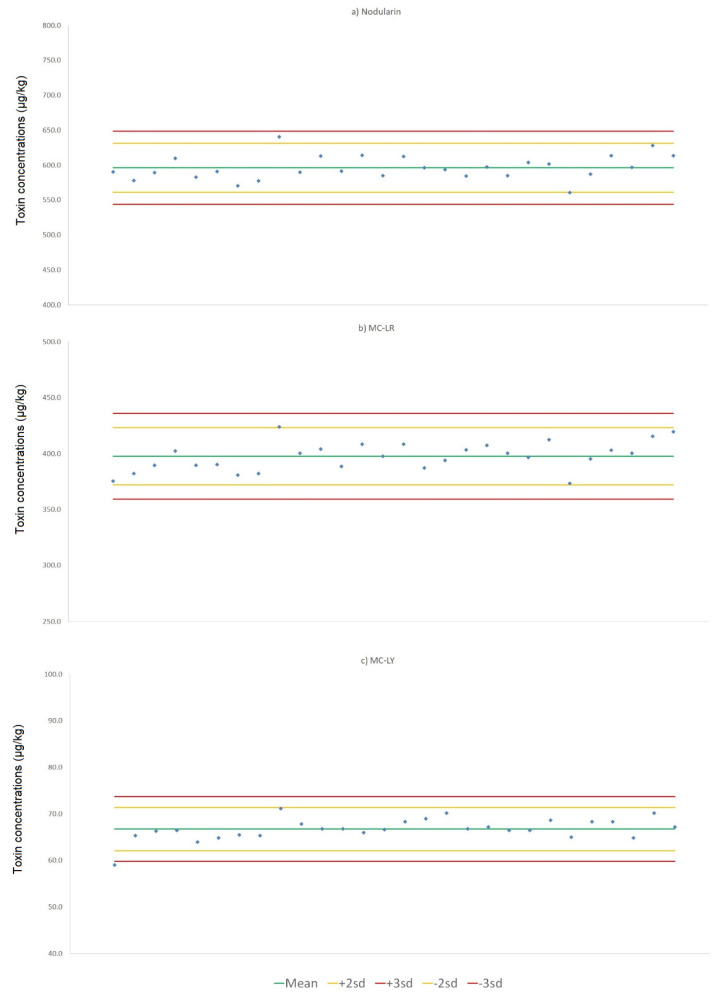

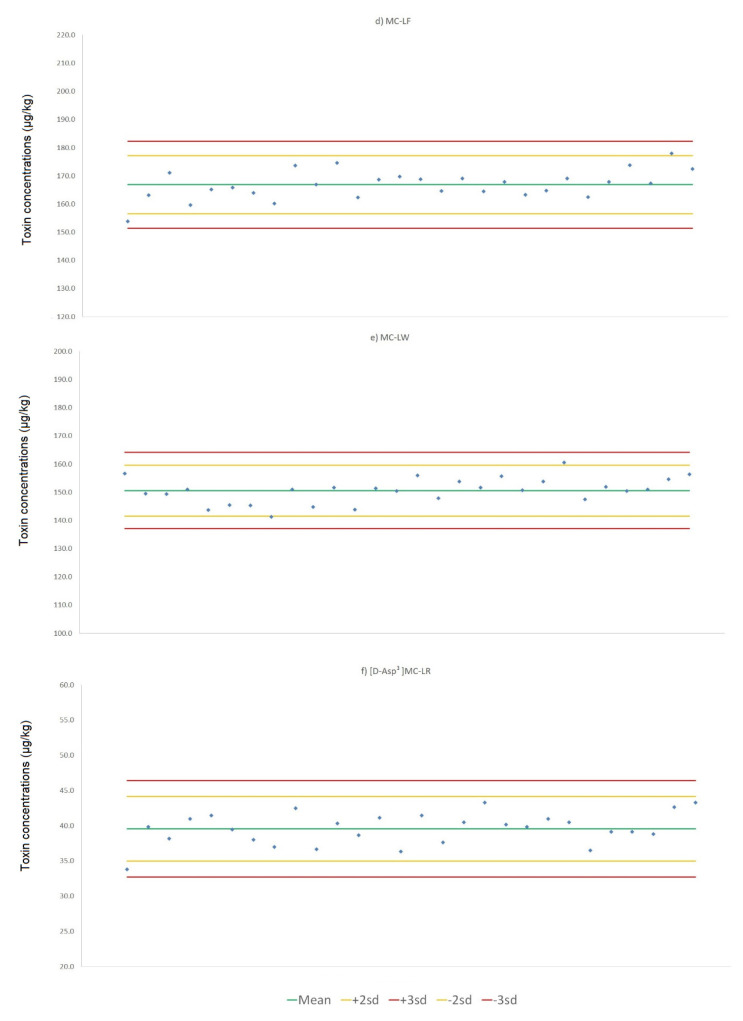
Cyanotoxin concentrations plotted in homogeneity control charts, showing scatter of individual test value duplicates (*n* = 20) (**a**) NOD-R (**b**) MC-LR (**c**) MC-LY (**d**) MC-LF (**e**) MC-LW (**f**) dmMC-LR ([D-Asp^3^]MC-LR/[Dha^7^]MC-LR).

**Figure A2:**
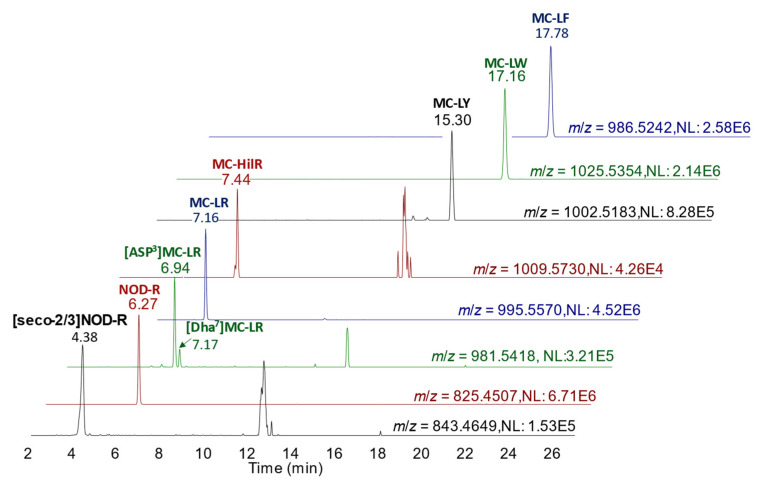
LC-HRMS chromatograms showing nodularin and microcystin profile identified in mussel tissue RM.

**Figure A3:**
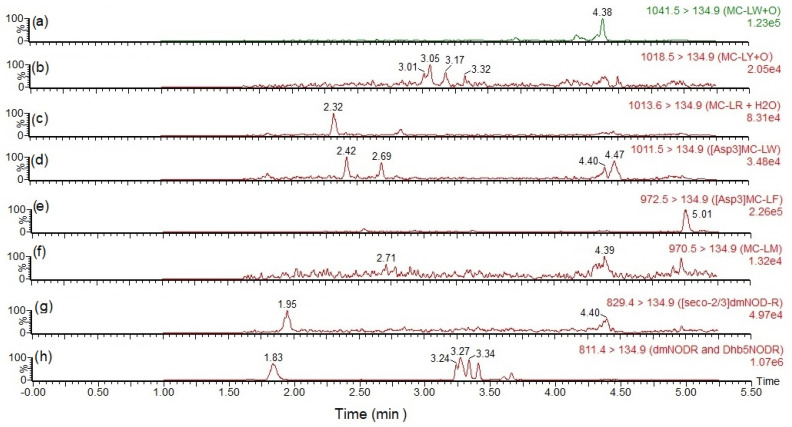
SRM chromatograms of minor cyanotoxin analogues obtained following LC-MS/MS analysis of mussel RM extracts highlighting detection of: (**a**) MC-LW + O (1041.5 > 134.9) (**b**) MC-LY + O (1018.5 > 134.9) (**c**) MC-LR + H_2_O (1013.6 > 134.9) (**d**) [D-Asp^3^]MC-LW (1011.5 > 134.9] (**e**) [D-Asp^3^]MC-LF (972.5 > 134.9) (**f**) MC-LM (970.5 > 134.9) (**g**) [seco-2/3]dmNOD (829.4 > 134.9] (**h**) dmNOD-R (811.4 > 134.9).

## Data Availability

Not applicable.

## Appendix A

**Table A1:** Summary of MS/MS conditions used for cyanotoxin determination.

Analyte	SRM Transitions ^a^	Cone Voltage (V)	Collision Energy (eV)
MC-RR	519.9 ^†^ > 134.9; 126.9; 102.8	30	30; 50; 70
NOD-R	825.5 > 135.1; 103.1	55	60; 100
MC-LA	910.1 > 135.1; 106.9	35	70; 80
[Dha^7^]MC-LR	981.5 > 135.0; 106.8	75	75; 80
[D-Asp^3^]MC-LR	981.5 > 134.9; 106.9	75	70; 80
MC-LF	986.5 > 213.0; 135.0	35	60; 65
MC-LR	995.6 > 135.0; 127.0	60	70; 90
MC-LY	1002.5 > 135.0; 106.9	40	70; 90
MC-HilR	1009.7 > 134.9; 126.9; 106.9	75	75; 90; 80
MC-LW	1025.5 > 134.9; 126.8	35	65; 90
MC-YR	1045.6 > 135.0; 126.9	75	75; 90
MC-HtyR	1059.6 > 134.9; 106.9	75	70; 90
MC-WR	1068.6 > 134.9; 106.9	80	75; 100
[seco-2/3]NOD	692.0 ^‡^ > 135.0; 107.0	55	54; 58
dmNOD-R	811.4 > 134.9	60	70
MC-LM	970.5 > 134.9	60	70
[D-Asp^3^]MC-LF	972.5 > 134.9	60	70
[D-Asp^3^]MC-LW	1011.5 > 134.9	60	70
MC-LY	1018.5 > 134.9	60	70
MC-LW	1041.5 > 134.9	60	70

^a^ precursor *m/z* > product *m/z* 1; product *m/z* 2; ^†^ Doubly charged precursor ion [M + 2H]^2+^; ^‡^ [M-NH_2_-135]+ as precursor ion [110].

## Appendix C

**Table A2:** Summary of MCs + NODs concentrations and total toxin concentrations (µg/kg wet weight) in individual crude extracts of LRM and raw material mussel samples following analysis by four techniques: LC-MS/MS, multihapten ELISA, Adda ELISA and MMPB for soluble and total toxins.

	RM Aliquots			Raw Exposed Mussel Tissues			Raw Unexposed Mussel Tissues		
	RM1	RM2	RM3	A	D	E	B	C	F
NOD-R	935	897	982	905	2764	2597	nd	nd	nd
[seco-2/3]-NOD	181	190	205	179	510	425	nd	nd	nd
MC-LR	530	492	465	420	1356	1265	nd	nd	nd
MC-LY	103	95	98	89	274	246	nd	nd	nd
MC-LF	231	260	223	191	600	542	nd	nd	nd
MC-LW	201	197	179	188	501	490	nd	nd	nd
MC-HilR	19	15	11	12	38	51	nd	nd	nd
dmMC-LR	62	51	55	49	134	111	nd	nd	nd
Sum of all MCs by LC-MS/MS	1146	1110	1031	949	2903	2705	nd	nd	nd
Sum of all NODs by LC-MS/MS	1116	1087	1187	1084	3274	3022	nd	nd	nd
Sum of all MCs + NODs combined by LC-MS/MS	2262	2197	2218	2033	6177	5727	nd	nd	nd
Multihapten ELISA	1458	1552	1262	294	2434	3782	nd	nd	nd
Adda ELISA	3788	3575	3350	893	3508	4258	nd	nd	nd
MMPB—soluble MCs + NODs	2156	2319	2766	1589	5994	5622	nd	nd	nd
MMPB—total MCs + NODs	3745	3969	3769	3422	11566	10921	nd	nd	nd

not detected (nd).

## Appendix G

A repeated measures ANOVA demonstrated that the lab method choice had a statistical effect on results (F = 12.699; *p* = 0.005). Table A3 summarises the linear regression characteristics between each pair of method results, showing similarities between some data sets (e.g., LC-MS/MS of MCs + NODs vs. MMPB soluble) but notable differences with others (e.g., Adda ELISA and MMPB Total). These relationships were assessed further using a Tukey post hoc analysis of pairwise differences (Table A4). Method differences assessed incorporated MCs + NODs by LC-MS/MS, both ELISA methods, MMPB soluble and MMPB total. Results highlighted statistical differences between the data quantified using multihapten ELISA and all other methods, as well as MMPB total with all other methods. No statistical differences were confirmed between LC-MS/MS and both the Adda ELISA and MMPB soluble concentrations, as well as between the Adda ELISA and MMPB soluble.

The multihapten ELISA correlated fairly well with the total MCs values determined by LC-MS/MS with higher overall results by ELISA, with a regression slope of 0.648 (r^2^ = 0.851) (Table A3). When incorporating NODs concentrations into the total LC-MS/MS results, the ELISA returned lower results in comparison to MCs + NODs combined but was still fairly well correlated (slope = 1.381, r^2^ = 0.839; Table A3). Conversely, the data returned using the Adda ELISA was higher on average than the total MCs + NODs combined (regression between ELISA and LC-MS/MS MCs + NODs: slope = 0.734; Tukey post hoc *p* = 0.9992), but with more variability in ratios between the two data sets resulting in a poorer correlation (r^2^ = 0.445).

Data generated following the MMPB analysis of soluble MCs + NODs quantified from solvent extracts compared closely with the total MCs + NODs results returned by LC-MS/MS with an excellent correlation described by the regression slope of 1.014 (r^2^ = 0.985) and no statistical difference following Tukey posthoc analysis (*p* = 1.0000). Correlations were lower between MMPB of extracts and both ELISAs, (Table A3), although there was no statistical difference between the MMPB soluble and Adda ELISA data (Tukey posthoc *p* = 0.9998; Table A4).

**Table A3:** Correlations between quantitative results returned for cyanotoxin-contaminated mussels (*n* = 6) using each method showing slope of linear regression with correlation coefficient (r^2^) in brackets.

Methods	LC-MS/MCs	LC-MS/NODs	LC-MS/MCs + NODs	Multihapten ELISA	Adda ELISA	MMPB Soluble	MMPB Total
LC-MS/MS MCs	1.000 (1.000)	0.858 (0.997)	0.463 (0.999)	0.648 (0.851)	0.359 (0.471)	0.469 (0.984)	0.232 (0.999)
LC-MS/MS NODs	-	1.000 (1.000)	0.537 (0.999)	0.732 (0.828)	0.375 (0.423)	0.545 (0.985)	0.270 (0.998)
LC-MS/MS MCs + NODs	-	-	1.000 (1.000)	1.381 (0.839)	0.734 (0.445)	1.014 (0.985)	0.502 (0.999)
Multihapten ELISA	-	-	-	1.000 (1.000)	0.766 (0.765)	0.543 (0.868)	0.259 (0.849)
Adda ELISA	-	-	-	-	1.000 (1.000)	0.337 (0.540)	0.137 (0.451)
MMPB Soluble	-	-	-	-	-	1.000 (1.000)	0.481 (0.986)
MMPB Total	-	-	-	-	-	-	1.000 (1.000)

**Table A4:** *p*-values from Tukey post hoc analysis of pairwise differences.

Methods	LC-MS/MCs + NODs	Multihapten ELISA	Adda ELISA	MMPB Soluble	MMPB Total
LC-MS/MS MCs + NODs	-	0.0047	0.9992	1.0000	0.0480
Multihapten ELISA	-	-	0.0080	0.0054	<0.0001
Adda ELISA	-	-	-	0.9998	0.0292
MMPB Soluble	-	-	-	-	0.0424
MMPB Total	-	-	-	-	-
